# Comparison of Photoacclimation in Twelve Freshwater Photoautotrophs (Chlorophyte, Bacillaryophyte, Cryptophyte and Cyanophyte) Isolated from a Natural Community

**DOI:** 10.1371/journal.pone.0057139

**Published:** 2013-03-19

**Authors:** Charles P. Deblois, Axelle Marchand, Philippe Juneau

**Affiliations:** Department of Biological Sciences, TOXEN, Ecotoxicology of Aquatic Microorganisms Laboratory, Université du Québec à Montréal, Montréal, Québec, Canada; University of New South Wales, Australia

## Abstract

Different representative of algae and cyanobacteria were isolated from a freshwater habitat and cultivated in laboratory to compare their photoacclimation capacity when exposed to a wide range of light intensity and to understand if this factor may modify natural community dominance. All species successfully acclimated to all light intensities and the response of phytoplankton to increased light intensity was similar and included a decrease of most photosynthetic pigments accompanied by an increase in photoprotective pigment content relative to Chl *a*. Most species also decreased their light absorption efficiency on a biovolume basis. This decrease not only resulted in a lower fraction of energy absorbed by the cell, but also to a lower transfer of energy to PSII and PSI. Furthermore, energy funnelled to PSII or PSI was also rearranged in favour of PSII. High light acclimated organisms also corresponded to high non-photochemical quenching and photosynthetic electron transport reduction state and to a low Φ'_M_. Thus photoacclimation processes work toward reducing the excitation pressure in high light environment through a reduction of light absorption efficiency, but also by lowering conversion efficiency. Interestingly, all species of our study followed that tendency despite being of different functional groups (colonial, flagellated, different sizes) and of different phylogeny demonstrating the great plasticity and adaptation ability of freshwater phytoplankton to their light environment. These adjustments may explain the decoupling between growth rate and photosynthesis observed above photosynthesis light saturation point for all species. Even if some species did reach higher growth rate in our conditions and thus, should dominate in natural environment with respect to light intensity, we cannot exclude that other environmental factors also influence the population dynamic and make the outcome harder to predict.

## Introduction

In aquatic environment, success of microalgae and cyanobacteria depends on their individual capacity to convert light into biochemical energy through photosynthetic light reactions and to transform carbon and nutrients into biomass. Because of the physical properties of water and the presence of suspended particles, available light intensity for photosynthesis is highly variable in freshwater habitat [1], [2]. During sunny days, light intensity at the surface of waterbodies can be high enough to induce photoinhibition and cellular damage to exposed photosynthetic organisms [3], [4], [5]. Simultaneously, only few meters below the surface (sometime less), light intensity become limiting for photosynthesis and may represent less than 1% of surface irradiance [2], [6]. In order to cope with such variability, photosynthetic organisms have developed an array of phenotypic adjustments including photoacclimation processes [2], [7], [8], [9]. Photoacclimation to low or high light environments involves mid to long term adjustments of the photosynthetic apparatus and includes down regulation and *de novo* synthesis of cell constituents such has photosynthetic and non-photosynthetic pigments, photosystems I and II (PSI and PSII), RUBISCO, as well as changes in cell ultrastructure [8], [10], [11], [12], [13]. When exposed to high light environments, photoacclimation responses of most algae and cyanobacteria include a decrease of photosynthetic pigments (chlorophylls and phycobiliproteins) combined with an increase in photoprotective carotenoids [14], [15], [16]. The decrease in chlorophyll *a* (Chl *a*) is normally associated to a decrease in the number of photosystems, while a decrease in accessory pigments (Chl *b*, *c*, *d*, and phycobiliproteins) is associated to a decrease in the size of the light harvesting complexes (LHC) [9]. Combined to an increase in carotenoid (Car) content relative to Chl *a*, these adjustments allow a reduction of the excitation pressure on the photosynthetic apparatus and protect the organism against light induced reactive oxygen species damages [17], [18], [19]. On the other hand, under light limiting conditions, algae and cyanobacteria adjust their cellular constituents to increase light absorption efficiency [7], [9], [20].

The wide diversity observed in phytoplankton is impressive, and may influence their light utilization efficiency. Interspecific variation of size between individual cells can reach many orders of magnitude from sub-micrometric picoplankton up to microplanktonic species [21], [22]. While many species remain single cells (e.g. *Chlamydomonas* sp., *Cryptomonas* sp., *Navicula* sp.), others grow into structured colonies (e.g. *Pediastrum* sp., *Pandorina* sp., *Volvox* sp., *Merismopedia* sp.), filaments (e.g. *Aphanizomenon* sp., *Anabaena* sp.) or more or less defined clusters of cells (e.g. *Microcystis* sp., *Sphaerocystis* sp., *Ankistrodesmus* sp.) [23], [24]. Colonial organization provides some benefits such as protection against grazing, but this characteristic also comes with inconvenience such as increased density inducing stronger sinking rate and increased self-shading leading to lower light availability [21], [25], [26]. Some of these species may also have flagella or vacuoles permitting them to move in the water column in order to optimize light harvesting [2]. This broad diversity influencing their light utilization is not limited to the morphological properties of phytoplankton, but can also be seen at the photosynthetic, biochemical or physiological levels such as distinct pigmentation [27]. Chlorophyll *a* has a crucial role in the photosystem reaction center (RC) core and in the light harvesting complexes of oxygenic phytoplankton, thus, this pigment is common to all species. Nevertheless, light harvesting capacity also differs between species because of variability in composition of pigments such as chlorophyll *b*, *c*, *d*, carotenoïds and phycobiliproteins [20], [28]. Thus the great diversity of phytoplankton characteristics influencing photosynthesis and light harvesting may influence the efficiency of photoacclimation processes.

Many lakes from the eastern Townships in Québec (Canada) are impacted on a periodic basis by cyanobacterial bloom apparitions [29]. This phenomenon is a visible consequence of changes in algal community equilibrium, but the factors influencing this dynamic are not fully understood. Since light is a factor that can modify algal community [1], [30], [31], comparing photoacclimation responses of various species may help to estimate if this factor contribute to the periodic community imbalance observed in these aquatic ecosystems. In this study, we isolated 12 species of phytoplankton belonging to different algal groups from a single algal assemblage of a temperate dimictic eutrophic lake. Species were selected for their different sizes and strategies to harvest light (pigments, movement) in order to compare their photoacclimation responses and to determine their active light range and photoacclimation capacity. We showed that the general photoacclimation processes among the studied species were similar, but the extent of the responses varies providing possible selective advantages to some species.

## Materials and Methods

### Sampling and cell culture

In mid-July of 2008, water from the euphotic zone of the Réservoir Choinière, (Eastern Townships, Québec, Canada) was collected and inoculated into bold basal medium (BBM) enriched with carbonate (25 mg L^-1^) and silicate (80 mg L^−1^) (BBMsi). Species that successfully grew in that medium were isolated and cultivated in laboratory. From the species initially isolated, 12 species were selected in order to have a diversity of algal groups (Chlorophyte, Bacillariophyte, Cryptophyte and Cyanophyte) and traits: colonial, unicellular, flagellates, buoyant and different cell sizes (see Table 1 for details). Throughout the experiment all species were grown in 125 ml of fresh BBMsi in 250 ml flasks and periodically (frequency depending on growth rate) transferred into fresh medium to maintain the cells in exponential growth phase which provides reproducible physiological characteristics. Periodic inoculum transfers also minimized dead cell accumulation which in any cases remained negligible as confirmed by monitoring, on a daily basis, the stability of the maximum PSII quantum yield (F_V_/F_M_) and by microscopic observations.

**Table 1:** Group, species names and relevant morphological and physiological characteristics including pigments for each of the 12 species selected for this study.

Groups	Species names	Codes	Characteristics	Major pigments
Chlorophyte	*Ankistrodesmus falcatus*	CHL1	Colonial (2–6 cells) non-motile elongate cells,	Chl *a*, *b*, *c*
	*Pandorina morum*	CHL2	Colonial (16 cells), flagelate, big size	Chl *a*, *b*, *c*
	*Oocystis lacustris*	CHL3	Single non-motile ovoid cell or small colony (2–3 cells).	Chl *a*, *b*, *c*
	*Pediastrum boryanum*	CHL4	Colonial, planar, non-motil	Chl *a*, *b*, *c*
	*Chlamydomonas snowii*	CHL5	Unicellular, flagelates	Chl *a*, *b*, *c*
Bacillariophyte	*Aulacoseira granulata* var. *angustissima*	BAC2	Elongate curvated filament of 2–3 cells, non-motil, very low pigmentation	Chl *a, b*
	*Fragilaria crotonensis*	BAC3	Colonial, non-motil	Chl *a, b*
Cryptophyte	*Cryptomonas obovata*	CRY1	Unicellular, flagelates	PE/Chl *a, d*
Cyanophyte	*Phormidium mucicola*	CYA1	Small 0.8 µm non-buoyant rod-like colony (up to 4 cells), toxic.	PC/APC/Chl *a*
	*Microcystis flos-aquae*	CYA2	Colony (non-mucilaginous), small spherical cells 2 µm diameter, buoyant, toxic.	PC/APC/Chl *a*
	*Aphanizomenon flos-aquae*	CYA3	Association of numerous buoyant filamentous colony, toxic.	PC/APC/Chl *a*
	*Anabaena spiroïdes*	CYA4	Filamentous colony, buoyant, toxic.	PC/APC/Chl *a*

The code is the abbreviation associated to the group and used in the figures.

Each species was acclimated for several weeks in an environmental growth chamber (MTR30, Conviron, Manitoba, Canada) with a light:dark cycle of 16: 8 at 21°C to seven light intensities:14, 43, 76, 191, 341, 583 and 1079 µmol photons (PAR) m^−2^ s^−1^ (measured with a US-SQS/L Micro quantum sensor, Heinz Walz GmbH, Effeltrich, Germany, in the center of the culture flask containing 125 mL BBMsi). Both fluorescent (cool white fluorescent tubes Philips F72T8/TL841/HO) and incandescent bulbs (Philips 60 W) were used and in our conditions (one growth chamber containing all cultures simultaneously) light quality was similar for all light intensities (confirmed by spectroradiometric measurements; HR2000 UV+Vis, Ocean Optics Inc, USA). All measurements were done on independent triplicates of fresh and healthy cultures. For each trial, a new culture was prepared from the previous one and was allowed to grow until maximum PSII quantum yield and similar cell conditions (F_0_, F_M_ and F_V_/F_M_) were attained. Moreover, using fixed signal gain and dark acclimation period during chlorophyll fluorescence measurements allowed to use the F_0_ as a proxy of cell concentration from day to day [32] and this method was used to estimate the growth rate for each trial.

### Chlorophyll fluorescence measurement

Induction curves (IC) were measured using WATER-Pulse-Amplitude-Modulated fluorometer (WATER-PAM) (Heinz Walz GmbH, Effeltrich, Germany) on 15 min dark adapted algal suspensions (3 mL) using standard IC protocol with the actinic light carefully matched to growth photon flux density (PFD). The PSII maximum and operational quantum yields (F_V_/F_M_ and Φ'_M_), the non-photochemical quenching (NPQ) and the unquenched fluorescence level (UQF_rel_) were calculated from each IC [33], [34]. For cyanobacteria, F_M_ was estimated at the end of each IC using 50 µM Diuron (DCMU) in presence of actinic light [35].

### Pigments determination

At each sampling, known volumes of culture were filtered under dime green light on GF/F filters (Whatman, USA) and kept frozen at −80°C until pigment and phycobiliprotein extraction and determination. Chlorophylls (Chls) and carotenoids (Car) were extracted 5 min in 4 mL of boiling methanol, rigorously vortexed 1 min and kept at −80°C for overnight extraction. Prior to measurement, the extract was filtered on GF/F and the optical density was read between 350 and 800 nm with a Cary 300 WinUV spectrophotometer (Varian, USA). The average OD from 750 to 800 nm was used to correct for sample turbidity. The concentrations of Chl *a*, *b*, *c* and *d* were estimated according to [36], while carotenoid concentration was estimated following [37]. Phycobilioproteins: phycocyanin (PC), allophycocyanin (APC), and phycoerythrine (PE), were extracted using 4 freeze-thaw cycles in 0.1 M potassium phosphate buffer (pH 6.8), sonicated on ice between the second and third cycle (2 watts for 1 min., sonic dismembrator model 100-Fisher Scientific, USA), and finally centrifuged at 5000× *g* for 15 min. The absorbance spectra of the supernatant was recorded between 500 and 700 nm using a Cary 300 WinUV spectrophotometer (Varian, USA) and pigment concentrations were calculated according to the equation given in [38].

### Cell division rate

The cell concentration and size were measured using a Multisizer III Coulter counter (Beckman Coulter Inc, Fullerton, USA) when cell morphology allowed it, while for the other species (colony, filament or non-spherical cells) a sample was fixed with Lugol solution for measurement and counting under a microscope. Species specific growth rate ( µ_d_) was calculated from these data and fitted to growing light intensity (PFD) with a classic 4 parameter PE curve model with photoinhibition [39]:



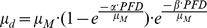
(1)


where α represents the initial slope of the curve, β is a light dependent inhibition constant and µ*_M_* is the theoretical maximum growth rate. From these coefficients, the secondary parameters: achieved maximal growth rate ( µ*_MAX_*) and its corresponding light intensity (E_M_^ µd^) were calculated [40].

### Biooptical measurements

All measurements were carried under dime green light. Sample were concentrated (5x) by gentle filtration on polycarbonate membrane filter 0.8 µm pore size (Millipore, USA) and resuspended in 3 mL of BBMsi directly in the measuring quartz cuvette. Cell lost on the filter was negligible. The *in vivo* light absorption spectrum of the concentrated solution (O.D. m^−1^) was measured (between 350 and 800 nm) with a Cary winUV spectrophotometer (Varian, USA) using the integrating sphere attachment. Immediately after this measurement, 10 µL of DCMU was added to the sample at a final concentration of 50 µM and the sample was maintained 1 minute under white light (500 µmol photons m^−2^ s^−1^) to eliminate any variable fluorescence. Following this treatment, the sample was immediately transferred in a CaryEclipse spectrofluorometer (Varian, USA) and the *in vivo* fluorescence excitation spectrum (400–700 nm) of the cell suspension was monitored at 730 nm. To avoid light scattering from the apparatus and cell sample, a long pass glass filter (RG695, Schott, AG, Mainz, Germany) was placed in front of the emission beam [20].

### Chl a specific absorption coefficient

The *in vivo* light absorption spectrum (O.D. m^−1^) was corrected for sample turbidity (average OD_750_800_) and converted to Chl *a* specific absorption coefficient a^*^_φ_(*λ*) (m^2^ mg Chl *a* ^−1^) according to eq. 2:



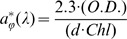
(2)


where 2.3 is the Log to Ln conversion factor, *d* is the cuvette path length (m^−1^) and *Chl* is the corresponding Chl *a* concentration (mg m^−3^). The fluorescence excitation spectra was quantum corrected using the dye Basic Blue 3 (4.1 g L^−1^) which corrects for instrument specific wavelength variation of the excitation beam intensity [41]. The corrected spectra was then scaled to a^*^_φ_ (*λ*) using the no-overshoot procedure to obtained the PSII Chl *a* specific absorption coefficient: *a* ^*^_PSII_ (λ) [20]. We averaged a^*^_φ_ (*λ*) and *a* ^*^_PSII_ (λ) between 400 and 700 nm or in the red band, between 670 and 680 nm, to obtain the light absorption coefficients specific to the whole cell (a^*^_φ_), to PSII and associated LHCII (*a* ^*^_PSII_), to Chl *a* in whole cell (a^*^_φ_ (red)) or to Chl *a* associated to PSII (*a* ^*^_PSII_ (λ)). High wavelength absorption efficiency (high a^*^_φ_(*λ*) or *a* ^*^_PSII_(λ)) is only significant when photons of that energy are available, hence, both coefficients were spectrally weighted according to the light spectrum *E*(λ) of the growth chamber. This correction was done by normalizing the *E*(λ) area to unity and by multiplying this dimensionless spectrum with a^*^_φ_(*λ*) or *a* ^*^_PSII_(λ) which yield the spectrally weighted (ā^*^_φ_(λ) and *ā* ^*^_PSII_(λ)) coefficient to be utilized in oxygen production estimates (see below).

### Oxygen production estimate

The best method to estimate oxygen production rate per chlorophyll unit (P_O2_^Chl^) using biooptical approach and chlorophyll fluorescence measurement was showed to be the method relying on *ā* ^*^_PSII_ to calculate light available to PSII photochemistry [42]. Therefore, we calculated the P_O2_^Chl^ data accordingly:



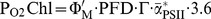
(3)


where *ā* ^*^_PSII_ represent light absorption specific to PSII (m^2^ mg chl *a* ^−1^) spectrally weighted to available light intensity and spectrum over the PAR range, PFD is the light intensity in the growth chamber ( µmol photons (PAR) m^−2^ s^−1^), Φ′_M_ is the PSII operational quantum yield, Г is the minimum theoretical quantum requirement of PSII in order to evolve one O_2_ molecule (0.25 O_2_ per electron) [43], and 3.6 convert second to hour (3600) and µmol to mmol giving the final dimension for P_O2_^Chl^ of mmol O_2_ mg chl *a* ^−1^ hr^−1^. For some analysis, P_O2_^Chl^ was converted to oxygen production rate per biovolume unit (P_O2_^ µm^) express in fmol O_2_ µm^−3^ hr^−1^. Each rate obtained for individual species at their specific growth light intensity was plotted against acclimation PFD (PE curve) and fitted to the waiting in-line function using eq. 4 [44]:



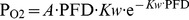
(4)


where, P_O2_ is oxygen production rate normalized to Chl *a* (P_O2_^Chl^) or biovolume (P_O2_^ µm^), *A* and *Kw* are scaling factors for the height of the curve and X-axis respectively and PFD was the light intensity ( µmol photons (PAR) m^−2^ s^−1^) in the growth chamber. From this function, we estimated the saturation (P_SAT_^Chl^/P_SAT_^ µm^) and maximum (P_M_^Chl^/P_M_^ µm^) rates of oxygen production and their corresponding light intensity (E_K_^Chl^/E_K_^ µm^ and E_M_^Chl^/E_M_^ µm^) following the equation presented in [44].

### Statistical analysis

All analysis were made in JMP 6.0 (SAS institute, USA) or GraphPad Prism software version 5.00 for Windows (GraphPad Software, San Diego California USA). The confidence interval (CI) at 95% was calculated for each coefficient in eq. 1 and eq. 4 using matrice inversion [44]. These coefficients value ± CI were compared by ANOVA and post Hoc Tukey Kramer mean comparison tests. Comparison between light limited and light saturated conditions was done for each species independently with student t-test, while two-way ANOVA was used to compare light limitation and light saturation responses between phylogenic groups [45]. Achieved maximal growth rate ( µ_MAX_) obtained for individual species was compared to the fit obtained for all species grouped (All species) using Dunnett's test (p<0.05) [46]. Subsequent comparison was done with Tukey Kramer test (p<0.05) to rank species in each subgroup (higher, equal or lower than All species µ_MAX_).

## Results

### Cell division rate and primary production

**Figure 1:**
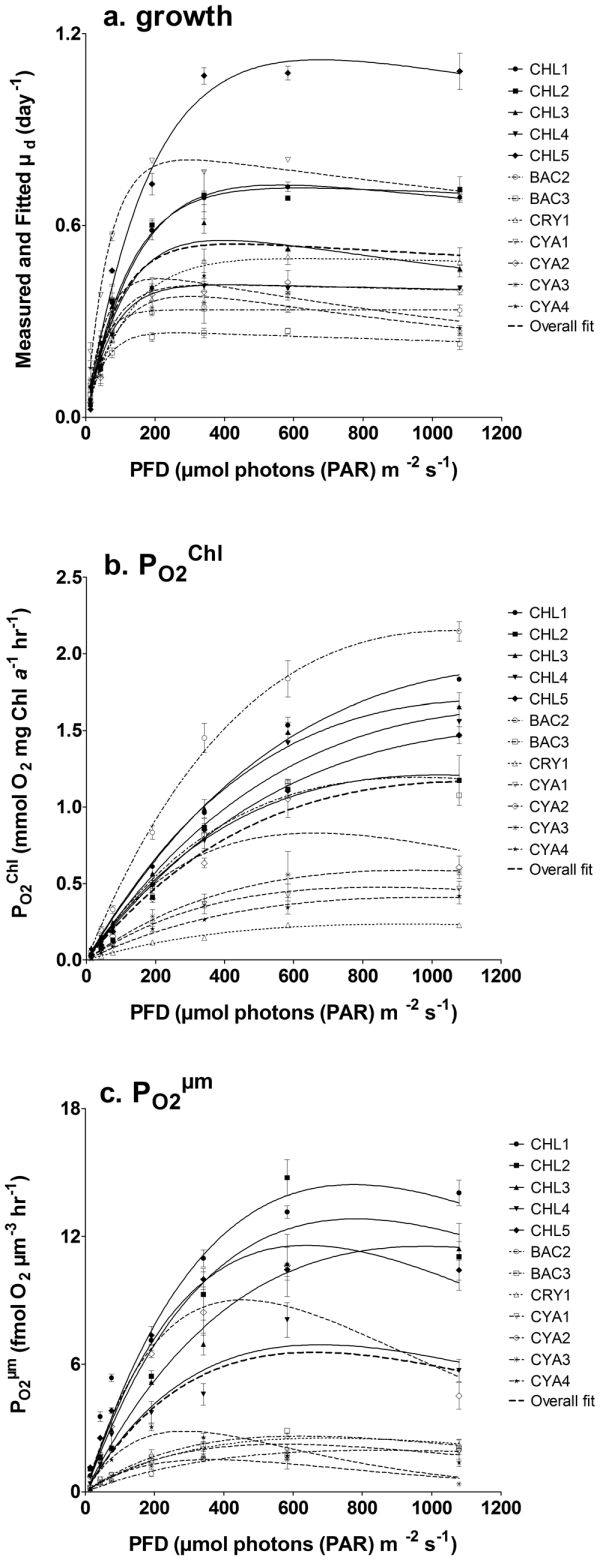
Responses of phytoplankton to light intensities; a. Cell division rate ( µ_d_). b. Oxygen production normalized to Chl *a* (P_O2_^Chl^) or c. to biovolume (P_O2_^ µm^) obtained at each growing light intensities of photoacclimated phytoplankton (see Table 1 for the species list). The corresponding fits for growth or photosynthesis versus irradiance curve (PE curve) were obtained using eq. 1 (for µ_d_) or eq. 4 (for P_O2_). Overall fit represents the result obtained for the whole data set.

Our results showed that all species successfully acclimated to all growth light conditions and that their specific cell division rates ( µ_d_ day^−1^) varied between 0.024 and 1.12 (Fig. 1a). Specific maximal growth rates ( µ_MAX_) of *Ankistrodesmus falcatus*, *Pandorina morum*, *Chlamydomonas snowii* and *Phormidium mucicola* were significantly higher compared to the overall averaged µ_MAX_ of 0.54(±0.05) using Dunnett's mean comparison (Table 2). In this group of fast growing species, mean comparison (Tukey HSD) showed that *C. snowii* reached the highest µ_MAX_, while there was no significant difference between *P. mucicola*, *A. falcatus* and *P. morum*. Above growth light saturation (between 200 and 400 µmol photons PAR m^−2^ s^-1^: data not shown), these fast growing species attained µ_d_ values that allowed population to double in a day or less ( µ_d_>0.693). Such level was not reached for any light conditions in other species of the present study (Fig. 1a). It is worth to notice that although *C. snowii* has the highest µ_d_ values under saturating irradiance, this species also has the lowest µ_d_ (0.024±0.002) when grown at 14 µmol photons (PAR) m^−2^ s^−1^, indicating that this light intensity was very close to its compensation point. Among the remaining species, *Aulacoseira granulata* var. *angustissima*, *Fragilaria crotonensis* and *Aphanizomenon flos-aquae* had significantly lower µ_MAX_ compared to the overall value and formed the group of slow growing species (Table 2). In this group, *F. crotonensis* had the lowest µ_MAX_ and was therefore the slowest growing species of this study.

**Table 2:** Achieved maximum growth rate ( µ_MAX_) and light intensity required to reach that rate (E_M_^ µd^) estimated from growth versus irradiance fit (R^2^ of each fit are presented) using eq. 1.

Species	E_M_^ µd^ ( µmol photons m^−2^ s^−1^)	Error	µ_MAX_ (day^−1^)	Error	R^2^ of Fit	p-value Dunnett's (for µ_MAX_)	Tukey HSD (for µ_MAX_)
*C. snowii* (CHL5)	585	25	1.12	0.14	0.98	<.0001	a
*P. mucicola* (CYA1)	279	5	0.80	0.03	0.98	0.0002	b
*A. falcatus* (CHL1)	503	30	0.73	0.08	0.96	0.0095	b
*P. morum* (CHL2)	588	39	0.72	0.08	0.95	0.0151	b
*O. lacustris* (CHL3)	340	2	0.55	0.06	0.91	1	a
*C. obovata* (CRY1)	605	39	0.50	0.05	0.94	0.9688	ab
*A. spiroïdes* (CYA4)	187	6	0.43	0.03	0.87	0.2497	ab
*M. flos-aquae* (CYA2)	369	64	0.41	0.06	0.73	0.1189	b
*P. boryanum* (CHL4)	403	19	0.41	0.02	0.97	0.1189	b
*A. flos-aquae* (CYA3)	252	6	0.38	0.05	0.81	0.0257	a
*A. granulata* (BAC2)	375	18	0.34	0.01	0.95	0.0032	a
*F. crotonensis* (BAC3)	255	20	0.26	0.02	0.90	<.0001	b
All species	401	29	0.54	0.05	0.48	1	-

The achieved maximal growth rate ( µ_MAX_) obtained for individual species was compared to the fit obtained for all species grouped (All species) using Dunnett's test (p<0.05). Subsequent comparisons with Tukey test (p<0.05) were done to rank species in each subgroup (higher, equal or lower than All species µ_MAX_). Presented error corresponds to the 95% confidence interval.

Light intensity required to reach µ_MAX_ (E_M_^ µd^) varied between 187(±6) and 605(±39) µmol photons m^−2^ s^−1^ for all species with an average of 401(±29) µmol photons m^−2^ s^−1^ (Table 2). The species showing the lowest E_M_^ µd^ were cyanophytes (except *Microcystis flos-aquae*) and the bacillariophyte *F. crotonensis*. Although they reached µ_MAX_ at similar light intensity, *F. crotonensis* was able to achieve constant growth rate above this point, while up to 35% growth inhibition was observed for the cyanophytes (except *M. flos-aquae*) (Fig. 1a). Growth inhibition was also observed for the cholorophyte *Oocystis lacustris* despite its high E_M_^ µd^. From all species, the cyanophyte *P. mucicola* was the best low light adapted organism since it achieved the highest µ_d_ at PFD below 191 µmol photons (PAR) m^−2^ s^−1^ (Fig. 1a). On the other hand, the best high light adapted organism was *C. snowii* because of its high µ_MAX_ and µ_d_ at light above 191 µmol photons (PAR) m^−2^ s^−1^.

Oxygen production (P_O2_^Chl^) estimates for all species varied between 0.008 and 2.239 mmol O_2_ mg Chl *a* ^−1^ hr^−1^ and the resulting PE curves depicted typical increases of photosynthetic activity in function of acclimation light intensity (Fig. 1b). When comparing the maximal oxygen production rate (P_M_^Chl^), our data showed significant differences between algal groups (Table 3). It was higher for the bacillariophytes (1.67±SD of 0.54 mmol O_2_ mg chl *a* ^−1^ hr^−1^) and chlorophytes (1.59±SD of 0.27 mmol O_2_ mg chl *a* ^−1^ hr^−1^), while it was lower for the cyanophytes with 0.58 (±0.19) mmol O_2_ mg chl *a* ^−1^ hr^−1^ and the cryptophyte with 0.23 (±0.02) mmol O_2_ mg chl *a* ^−1^ hr^−1^ (Table 3). Oxygen production was also normalized to biovolume (P_O2_^ µm^ unit: fmol O_2_ µm^−3^ hr^−1^) and allowed to compare species with respect to biomass, provided that cellular volume ( µm^3^) was a good proxy of species biomass [47]. Fitting P_O2_^ µm^ with eq. 4, yielded different parameter estimates (P_M_^ µm^ and E_M_^ µm^) compared to the result obtained from P_O2_^chl^ data (Fig. 1b and 1c). When comparing the maximal oxygen production rate per biovolume (P_M_^ µm^), our data showed that chlorophytes reached the highest value with an average of 11.46(±2.82) fmol O_2_ µm^−3^ hr^−1^ (Table 3). Comparatively, it was up to 10 times lower for bacillariophytes, cryptophytes and cyanophytes (except *M. flos-aquae*) and varied between 1.50 and 2.84 fmol O_2_ µm^−3^ hr^−1^ (Table 3). The light intensities at which P_M_^Chl^ and P_M_^ µm^ were achieved (E_M_^Chl^ and E_M_^ µm^) were also different and our data showed that E_M_^ µm^ was significantly lower for all species (Table 3). For E_M_^Chl^, it varied between 660 and 1376 µmol photons m^−2^ s^−1^, while for E_M_^ µm^ it varied between 287 and 979 µmol photons m^−2^ s^−1^ (Table 3).

**Table 3:** Calculated parameters and associated errors (95% interval) of PE curves fitted (see also Fig. 1.1b and c) using waiting in line function (eq. 4) for each species or combined all data (All species).

	P_M_^Chl^ (mmol O^2^ mg Chl *a* ^−1^ hr^−1^)	error	E_M_^Chl^ ( µmol photons m^−2^ s^−1^)	error	P_M_^ µm^ (fmol O^2^ µm^−3^ hr^−1^)	error	E_M_^ µm^ ( µmol photons m^−2^ s^−1^)	error
*A. falcatus* (CHL1)	1.91	0.09	1376	112	14.44	1.29	774	74
*P. morum* (CHL2)	1.21	0.16	1022	174	12.82	2.02	780	133
*O. lacustris* (CHL3)	1.69	0.13	1169	131	11.54	0.94	979	101
*P. boryanum* (CHL4)	1.64	0.17	1335	225	6.92	1.15	678	113
*C. snowii* (CHL5)	1.50	0.09	1361	131	11.58	0.81	639	43
*A. granulata* (BAC2)	2.15	0.15	1061	98	1.95	0.42	851	211
*F. crotonensis* (BAC3)	1.19	0.12	961	152	2.61	0.27	622	62
*C. obovata* (CRY1)	0.23	0.02	926	77	2.52	0.32	697	89
*P. mucicola* (CYA1)	0.48	0.07	843	148	2.22	0.32	570	76
*M. flos-aquae* (CYA2)	0.83	0.15	660	119	9.95	2.14	480	89
*A. flos-aquae* (CYA3)	0.59	0.12	951	232	1.50	0.29	363	57
*A. spiroïdes* (CYA4)	0.41	0.06	1002	176	2.84	0.42	287	33
All species	1.17	0.18	1129	243	6.56	1.24	660	123

Presented error corresponds to the 95% confidence interval.

**Figure 2:**
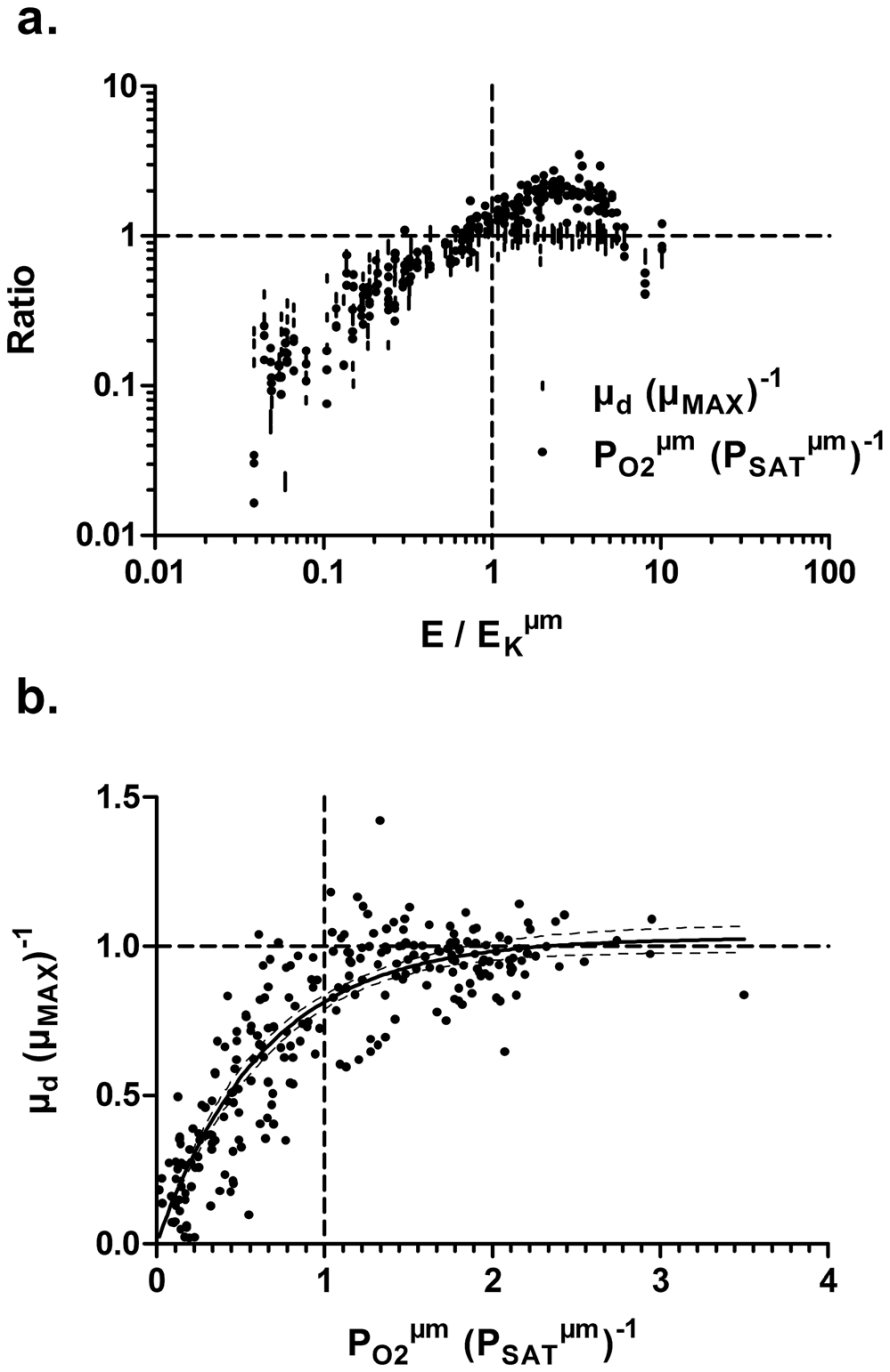
Responses of photosynthesis to light intensities; a. Oxygen production per biovolume (P_O2_^ µm^) relative to oxygen production at saturation (P_SAT_^ µm^) and achieved growth rate ( µ_d_) relative to maximal growth rate ( µ_MAX_) obtained for a gradient of growing light intensity (E) normalized to saturating light intensity of oxygen production (E_K_^ µm^); b. relationship between obtained growth rate µ_d_ normalized to µ_MAX_ and oxygen production per biovolume (P_O2_^ µm^) normalized to oxygen production at light saturation (P_SAT_^ µm^). For both panels, the dashed line was set to 1 for all ratios and by definition corresponds to the point where the achieved value ( µ_d_, P_O2_^ µm^ or E) equals the normalized coefficient value∶ µ_MAX_, P_SAT_^ µm^ or E_K_^ µm^.

Species differences in PE curve inevitably introduce noise when comparing light dependent variables such as pigments [9]. This problem was accounted for by dividing growth PFD (E) to the photosynthesis light saturation point per biovolume (E_K_^ µm^) obtained for each species. This variable (E∶E_K_^ µm^) was utilised to compare light dependent variables between species and allowed to form 2 groups corresponding to light limitation (E∶E_K_^ µm^<1) or saturation (E∶E_K_^ µm^>1) [9]. We also extended this approach to other variables: P_O2_^ µm^ using the rate of photosynthesis at saturation P_SAT_^ µm^ and µ_d_ using µ_MAX_ (Fig. 2a and 2b). Comparing these variables showed that most species, regardless of their phylogeny, followed very similar trend with respect to saturation of photosynthesis and cell division rate (Fig. 2a). The relationships between µ_d_∶ µ_MAX_ and E∶E_K_^ µm^ and between P_O2_^ µm^∶P_SAT_^ µm^ and E∶E_K_^ µm^ showed for all species that cell division reached µ_MAX_ ( µ_d_∶ µ_MAX_ = 1) and that photosynthesis reached saturation (P_O2_^ µm^∶P_SAT_^ µm^ = 1) at E_K_^ µm^ (Fig. 2a). Similar results were obtained when comparing the relationship between P_O2_^ µm^∶P_SAT_^ µm^ and µ_d_∶ µ_MAX_ (Fig. 2b) and we observed that below saturation of photosynthesis, growth rate linearly increased, while it stabilized to µ_MAX_ above photosynthetic saturation (Fig. 2b).

### Pigment content

**Figure 3:**
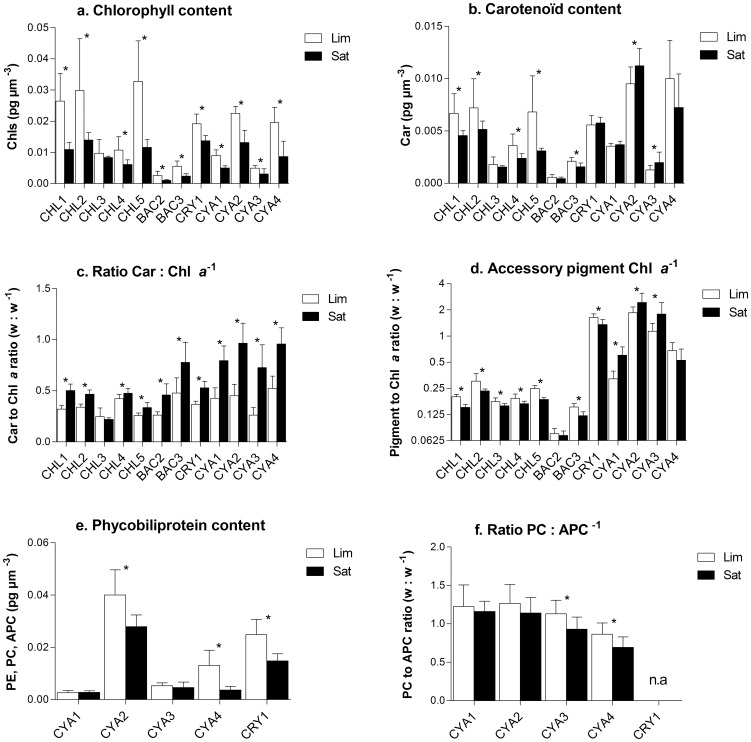
Comparison of the average pigment content normalized to biovolume (pg µm^−3^) or the average pigment ratios obtained for each species grown under photosynthetic light limiting (Lim) or light saturating (Sat) conditions; a. total chlorophyll content, b. carotenoid content, c. Car to Chl *a* ratio, d. sum of accessory pigments (Chl *b*, *c*, d and phycobiliproteins), e. phycobiliprotein content for species having these pigments and f. phycocyanin (PC) to allophycocyanin (APC) ratio in cyanophytes. ^*^ Significant difference between treatment obtained for each species using t-test (p<0.05). See Table 1 for species list.

For all species, Chl *a* content was higher in phytoplankton exposed to light limiting conditions (PFD < E_K_^ µm^) compared to saturating conditions (PFD > E_K_^ µm^), although it was not significant for *O. lacustris* (Fig. 3a). Our data also showed that bacillariophytes have less Chl *a* compared to most species. There was great variability in photoprotective carotenoid response since it tended to increase above light saturation for most cyanophytes but it decreased for most chlorophytes and bacillariophytes or remained unchanged in cryptophyte and one cyanophyte (Fig. 3b). Despite these different responses, the Car to Chl *a* ratio followed a similar trend for all species (except *O. lacustris*) and was significantly higher above light saturation (Fig. 3c). The sum of accessory pigments (PC, APC, PE, Chl *b*, Chl *c* and Chl *d*) relative to Chl *a*, reflecting the size of the light harvesting antennae, also varied in function of light intensity. This ratio was higher under light limiting conditions, while it was lower when phytoplankton grew under light saturating conditions (it was the opposite for cyanophytes) (Fig. 3d). In cyanophytes and cryptophyte this ratio was generally higher compared to the other species because of phycobiliproteins, important for light harvesting in these species [48]. Our results showed that phycobiliproteins content decreased above light saturation for *M. flos-aquae*, *Anabaena spiroïdes* and *Cryptomonas obovata* and that the ratio of PC to APC also decreased for both filamentous species, *A. flos-aquae* and *A. spiroïdes* (Fig. 3e and 3f). Finally, we observed that phycobiliproteins (PC and APC or PE) decreased on average by 30% from low to high light, while Chl *a* decreased by 40% for the same conditions.

### Biooptical characteristics

**Figure 4:**
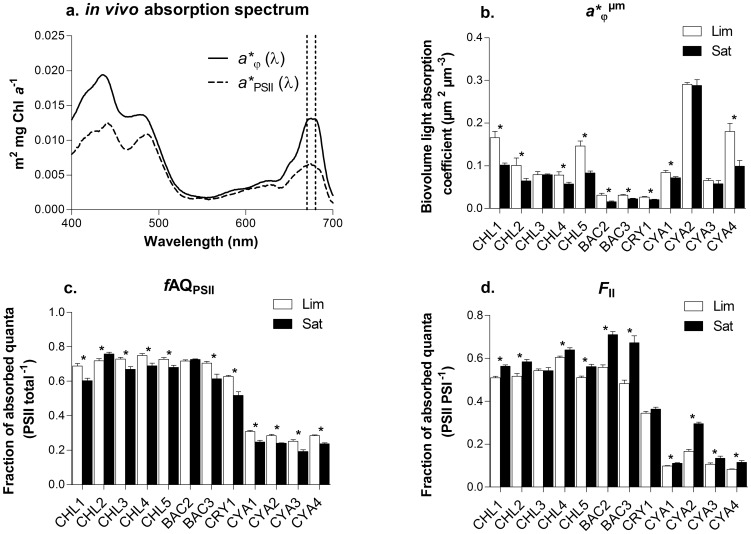
Responses of biooptical variables to light intensities; a. example of *in vivo* Chl *a* absorption spectrum (a*_φ_ (λ) and a*_PSII_ (λ)) obtained for *A. falcatus* acclimated to 76 µmol photons m^−2^ s^−1^. Averaging a*_φ_ (λ) or a*_PSII_ (λ) over the whole spectrum (400 to 700 nm) yielded to a*_φ_ and a*_PSII_ respectively, while averaging the coefficient in the red band (670 to 680 nm) yielded to a*_φ_ (red) and a*_PSII_ (red) respectively. Other panels, comparison of averaged biooptical data obtained for each species grown under photosynthetic light limiting (Lim) or light saturating (Sat) conditions where b. is the averaged light absorption coefficient normalized to biovolume, c. the fraction of absorbed quanta to PSII (*f*AQ_PSII_ = a*_PSII_/a*_φ_) and d. the fraction of absorbed quanta associated to PSII relative to PSI (F_II_ = a*_PSII_ (red)/a*_φ_ (red)). * Significant difference between treatment obtained for each species using t-test (p<0.05). See Table 1 for species list.

Modifications of pigments content induced by photoacclimation processes also modified the biooptical properties of individual cells. For all conditions and studied species, the *in vivo* Chl *a*-specific light absorption coefficient in the red (a^*^ *_φ_* (red)) varied between 0.002 and 0.025 m^2^ mg chl *a* ^−1^ with the lowest value(<0.005) obtained for *C. obovata* (Table 4). When averaged over the whole spectrum, the corresponding light absorption coefficient reflecting total light absorption by pigments (*a* ^*^ *_φ_*) or specific to PSII (*a* ^*^_PSII_), varied between 0.001 and 0.032 m^2^ mg Chl *a* ^−1^ and 0.001 and 0.013 respectively (Table 4 and e.g. Fig. 4a). For most species, these coefficients were higher under photosynthetic light saturation conditions, except for *C. obovata* and *O. lacustris* (for *a* ^*^_φ_, *a* ^*^_PSII_ and *a* ^*^_φ_ (red)) and *A. granulata* (for *a* ^*^_φ_ (red)) (Table 4). Increased light absorption efficiency in high light, as presented here, was counterbalanced by the lower Chl *a* content above light saturation (Fig. 3a). In fact, when normalizing light absorption coefficients to the Chl *a* content per biovolume, correcting for changes in Chl *a* quotas due to photoacclimation processes, our data showed a decrease of all coefficients (*a* ^*^_φ_, *a* ^*^_PSII_ and *a* ^*^_φ_ (red)) above light saturation (e.g. *a* ^*^_φ_^ µm^ in Fig. 4b; data not shown for other coefficients). The fraction of light absorption associated to PSII and LHCII relative to light absorption by the whole cell (*f*AQ_PSII_) decreased by 8.9 to 26.1% for most species following high light acclimation (Fig. 4c). On the other hand, the cellular fraction of Chl *a* associated to PSII relative to that associated to PSI (F_II_) was found to increase by 5.7 to 76.9% depending on species (Fig. 4d). Regardless of the light conditions, we also found that a significantly higher fraction of light absorption was directed toward PSII (*f*AQ_PSII_) in chlorophytes (0.73±0.05) and bacillariophytes (0.72±0.05) compared to cryptophyte (0.63±0.08), while it was much lower (0.33±0.06) in cyanophytes (Fig. 4c). For all light conditions, cyanophytes were also characterized by very low F_II_ (0.14±0.07) compared to cryptophyte (0.35±0.01), chlorophytes (0.56±0.04) and bacillariophytes (0.61±0.10).

**Table 4:** Averaged data (% CV) obtained and compared between light limiting (Lim) and light saturating (Sat) intensity for photosynthesis of the 12 studied species.

	*a* *_φ_		*a* *_PSII_		*a* *_φ_ (red)	
	Lim	Sat	Lim	Sat	Lim	Sat
*A. falcatus* (CHL1)	0.008 (19)	0.011 (8)*	0.005 (16)	0.007 (3)*	0.012 (19)	0.015 (4)*
*P. morum* (CHL2)	0.004 (17)	0.006 (14)*	0.003 (16)	0.004 (15)*	0.006 (13)	0.008 (14)*
*O. lacustris* (CHL3)	0.011 (24)	0.011 (4)	0.008 (27)	0.008 (7)	0.016 (20)	0.017 (4)
*P. boryanum* (CHL4)	0.009 (16)	0.012 (15)*	0.007 (16)	0.008 (10)*	0.012 (15)	0.014 (10)*
*C. snowii* (CHL5)	0.006 (17)	0.009 (12)*	0.004 (16)	0.006 (9)*	0.009 (17)	0.013 (9) *
*A. granulata* (BAC2)	0.014 (7)	0.016 (8)*	0.010 (6)	0.012 (9)*	0.021 (8)	0.022 (10)
*F. crotonensis* (BAC3)	0.007 (15)	0.012 (33)*	0.005 (12)	0.007 (20)*	0.009 (12)	0.012 (13)*
*C. obovata* (CRY1)	0.002 (18)	0.002 (10)	0.001 (18)	0.001 (17)	0.002 (18)	0.002 (14)
*P. mucicola* (CYA1)	0.010 (16)	0.016 (17)*	0.003 (13)	0.004 (8)*	0.012 (13)	0.014 (8)*
*M. flos-aquae* (CYA2)	0.014 (9)	0.025 (20)*	0.004 (6)	0.006 (17)*	0.015 (6)	0.017 (12)*
*A. flos-aquae* (CYA3)	0.014 (19)	0.023 (33)*	0.003 (15)	0.004 (26)*	0.014 (13)	0.017 (17)*
*A. spiroïdes* (CYA4)	0.010 (14)	0.013 (16)*	0.003 (10)	0.003 (13)*	0.011 (8)	0.013 (8)*

Pigment absorption was averaged over the whole light absorption spectrum (400 to 700 nm) for whole cell (a*_φ_) or specific to PSII (a*_PSII_), or was averaged in the red band (670 to 680 nm) for Chl *a* specific absorption (a*_φ_ (red)).

* Significantly different by t-test (p<0.05), unequal variance assumed.

### Photosynthetic electron transport and quantum requirement

**Figure 5:**
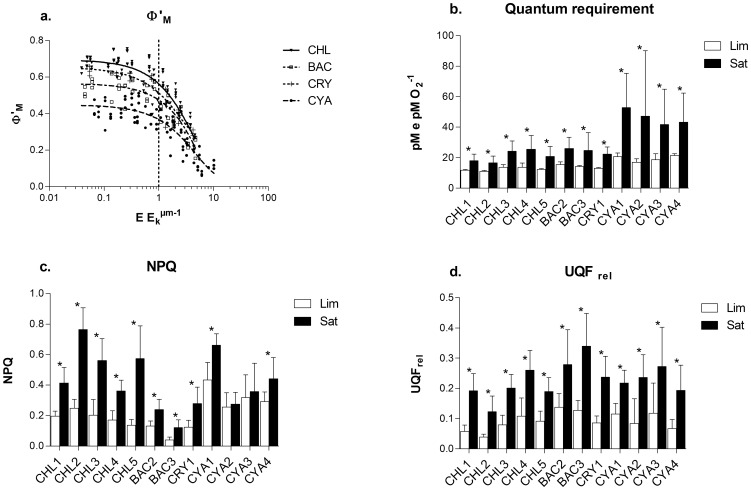
Responses of chlorophyll fluorescence parameters to light intensities; a. Group specific relationship between PSII operational quantum yield (Φ'_M_) and growth light intensity normalized to photosynthetic light saturation point (E_K_^ µm^). Other panels, comparison of averaged chlorophyll fluorescence paramaters obtained for each species grown under photosynthetic light limiting (Lim) or light saturating (Sat) conditions where b. is the quantum requirement, c. the non-photochemical quenching (NPQ) and d. the relative unquenched fluorescence parameter (UQF_Rel_). * Significant difference between treatment obtained for each species using t-test (p<0.05). See Table 1 for species list.

In this section, we compared the effect of photoacclimation on photosynthesis through photosynthetic electron transport, light utilisation and dissipation and quantum requirement (QR). Decrease in the PSII operational quantum yield (Φ'_M_) was observed with increasing light intensity for the 12 studied species (Fig. 5a). Our data showed that Φ'_M_ remained stable under light limiting conditions (E∶E_K_^ µm^<1) and decreased by 54 to 85% at light intensities above E_K_^ µm^ (Fig. 5a). The averaged Φ'_M_ was the highest for chlorophytes (0.19–0.75) followed by cryptophyte (0.28–0.66) and bacillariophytes (0.15 to 0.63), while it was the lowest in cyanophytes (0.06–0.51) (Fig. 5a). The decrease of Φ'_M_ under high light conditions also affected the number of photons required to evolve 1 O_2_ molecule (oxygen quantum requirement: QR). As seen, QR increased (from 149 up to 279%) for all species when light intensity increased above saturation level (Fig. 5b). Under light limiting conditions, where Φ'_M_ was the highest, the QR was on average 13.2±1.4 mol e mol O_2_^−1^ for all algal species, while it was higher in cyanophytes with 19.4±2.0 mol e mol O_2_^−1^. Furthermore, we noticed that the decrease of Φ'_M_ and the concomitant increase in QR were accompanied by an increase in non-photochemical quenching (NPQ) and in the level of unquenched fluorescence (UQF_rel_) (Fig. 5c and 5d). Group comparison showed that NPQ was higher in cyanobacteria (0.38±SE 0.02) and chlorophytes (0.33±SE 0.02) compared to cryptophyte (0.19±SE 0.02) and bacillariophytes (0.13±SE 0.01) and was surprisingly low in the latter group. We also demonstrated that NPQ tended to increase from limiting to saturating light conditions (average 220%) although that tendency was not significant for two of the cyanophytes studied in which NPQ was constant (Fig. 5c). Similarly, the unquenched fluorescence level (UQF_rel_), which is proportional to the redox state of the photosynthetic electron transport chain [34], increased (average 255%) for all species between limiting and saturating light conditions (Fig. 5d). When averaged over all light conditions, the highest UQF_rel_ value was measured in bacillariophytes (0.21±SE 0.01) followed by cyanophytes (0.17±SE 0.01) and cryptophyte (0.15±SE 0.02), while for chlorophytes the value (0.12±SE 0.01) was significantly lower.

## Discussion

### Effect on growth

In this study, we showed that chlorophytes, bacillariophytes, cryptophyte and cyanophytes successfully acclimate and grow under a wide range of light conditions (from 14 to 1079 µmol photons m^−2^ s^−1^) when given proper acclimation period. As expected, the achieved growth rate increased with light intensity and reached its maximal value between 187 and 605 µmol photons m^−2^ s^−1^ depending on the species. Above that point, growth inhibition was observed for most cyanophytes, corresponding to the response of low light adapted organisms [7], [30], but also for *O. lacustris*, while growth of other species remained unaffected by high light up to 1079 µmol photons m^−2^ s^−1^. Photoinhibition observed for *O. lacustris* was not surprising since we have shown low pigment plasticity and overall low photoacclimation driven responses (pigments, biooptic, photosystem ratio) for that species. As expected, this lack of response under high light conditions resulted in suboptimal growth [7], [17]. Although not mandatory, our growth data suggested that being chlorophyte, flagellate and/or small organism are characteristics allowing higher than average growth rate (Table 2). Conversely, most of the species presenting low growth rates (*A. granulata*, *F. crotonensis* or *A. flos-aquae*) were colonial or filamentous and had large cell as seen by their high averaged biovolume of 484 to 3593 µm^3^. It is well admitted that usually larger organisms have slower growth rates compared to smaller organisms due to their cell metabolism and higher package effect [21], [49]. However, we noticed some exceptions to that trend as seen with *M. flos-aquae* and *P. morum*. For *M. flos-aquae*, we have measured low growth rate despite its small size(±27 µm^3^), but this species also formed colonies. Another exception was observed for the colonial species *P. morum* for which high growth rate was measured despite its large size. For that species, individual cells are flagellated and we observed that their motion can actively position the colony in relation to available light and this may contribute to optimize its growth [27], [50]. Knowing that capacity to acclimate to light and morphological characteristics are important to determine growth [24], [31], our results tend to demonstrate that size, which affects the light absorption efficiency (package effect), and aptitude to movement (to optimize light harvesting) were relevant factors.

### Pigment acclimation

Because light can be damaging for photosystems, by causing oxydative stress to individual cell, and is indispensable as a source of energy, phytoplankton capacity to acclimate to a limitation or excess in photon flux is critical [12]. In this study, we showed that most species presented similar response to light acclimation, but to varying degrees. We found a decrease of photosynthetic pigment content (chlorophylls and phycobiliproteins) in all species following high light acclimation confirming previous observations [2], [8], [9], [13]. In most species, this decrease was accompanied by a decrease in carotenoid content and in the size of the light harvesting antennae as seen by lower accessory pigments to Chl *a* ratio (Fig. 3b and 3d). In cyanophytes, we observed a small reduction in the size of PBS under light saturation, but the proportion of PBS relative to Chl *a* increased suggesting an increase of LHC antenna size in that group (see below for more details). Lowering pigment content and antenna size is a typical response of high light acclimated cells [51]. These responses directly decrease the number of photons absorbed by the LHCs and decrease energy transfer to PSII and PSI RCs [51]. This adjustment results in a lower excitation pressure on the photosynthetic apparatus and is essential to minimize photoinhibition and cell damage induced by oxidative stress [52], [53]. In low light environment, and as observed in this study, these pigment modifications also worked in the opposite direction. In fact, increased pigmentation and antenna size allowed to maximize light harvesting and thus, alleviated the energy deficit caused by surrounding light scarcity [7], [9]. The light dependent variation in photosynthetic pigment content described here, was accompanied by modifications of the proportion of photoprotective pigments (Car) with respect to Chl *a* (Fig. 3c). This ratio was the highest under light saturation condition for all species and corresponded to previous finding showing that high Car to Chl *a* ratio increases protection against excess photon flux by allowing light energy dissipation through NPQ processes [13], [54], [55].

For all light conditions, cyanobacteria and cryptophyte had significantly more accessory pigments relative to Chl *a* and that was attributed to the presence of phycobiliproteins reflecting the dominance of these pigments for light harvesting in these species [2], [47], [56]. Surprisingly, this ratio was significantly higher under saturating light condition for three cyanobacteria, while it was lower in the other tested species of this study and others [9], [57], [58]. Since this increase was accompanied by a decrease of Chl *a* and PBS individually, it indicates that when acclimated to high light, these species favour light harvesting through PBS relative to Chl *a*. Phycobilisomes are highly mobile pigment complexes that can unbind from the RC core when exposed to high irradiance and thus prevent energy funnelling under excess light condition [59], [60]. Furthermore, previous studies have shown that in some cyanophytes, orange carotenoids interact with PBS when exposed to high light intensity in order to induce dissipation of excess energy [61], [62], [63]. Thus, the observed increase of PBS relative to Chl *a* may help to protect against high light as a complementary mechanism to energy dissipation through carotenoids. We can also hypothesis that favouring PBS over carotenoids is a strategy allowing higher light harvesting flexibility for organisms suddenly exposed to a lower light environment.

### Biooptical acclimation

Analysis of the biooptical data showed that changes in pigment content and ratio reported here successfully modified light harvesting efficiency and energy allocation between PSI and PSII (Fig. 4 and Table 4). For most species, the Chl *a* specific light absorption coefficients (*a* ^*^_φ_, *a* ^*^_φ_ (red) and *a* ^*^_PSII_) significantly increased with light intensity. This increase was important (reaching up to 169%) for all species with minor exceptions (*O. lacustris*, *A. granulata* and *C. obovata*) and as was found previously, this was related to an increase in light absorption efficiency for high light acclimated cells [20]. This counterintuitive result may be attributed to an increased light absorption of Car (relative to Chl *a*) and assigned to Chl *a* in a* calculation, but also to a lower pigment packaging (reduced self-shading) in high light acclimated cells [2], [20], [64], [65], [66]. According to our results, both phenomena occurred in our conditions since we observed an increase in the Car to Chl *a* ratio and the *a* ^*^_φ_ (red) values never reached 0.033 m^2^ mg Chl *a* ^−1^ (max value obtained was 0.025 m^2^ mg Chl *a* ^−1^, see also Table 4) and this value is expected to be close to the absorption coefficient of Chl *a* embedded in thylakoid membrane without any package effect [64]. Nevertheless, the higher light absorption efficiency observed for high light cells was mitigated by a lower content in Chl *a* per biovolume (Fig. 3a). When taking that variable into account, we observed that the biovolume specific absorption cross section coefficient (*a* ^*^_φ_^ µm^) decreased or remained stable as can be expected following acclimation to high light [2], [20], [48]. Very similar results and conclusions were drawn for PSII specific absorption coefficient (*a* ^*^_PSII_) and Chl *a* absorption in the red (*a* ^*^_φ_ (red)) indicating that following high light acclimation, energy directly associated to Chl *a* and PSII tended to decrease (in most cases) or remained stable on a biovolume basis. These modifications observed under high light conditions minimized the excitation pressure on the photosynthetic apparatus despite the increased light availability [18].

Comparison in the partition of harvested energy showed that above light saturation of photosynthesis, a lower proportion of intercepted photon was directed toward PSII in almost all species (Fig. 4c). This can be explained by the increase of Car to Chl *a* ratio and associated increased proportion of energy dissipation through heat by carotenoids and/or PBS uncoupling in cyanophytes. Our data also showed that energy balance between PSII and PSI was modified under high light, since a higher fraction of the energy was associated to PSII compared to PSI (Fig. 4d). Thus, it indicates that photoacclimation process did not only decrease PSII and PSI excitation pressure under higher light intensity, but it also redirected light absorption toward PSII. This rebalance of energy between the photosystems is necessary to prevent any excess energy to one of the photosystems (minimize excitation pressure) and to optimize electron flow between photosystems [11], [17], [18], [67].

### Photoacclimation and photosynthesis

Our data clearly showed that the photosynthetic activity of PSII was also affected by photoacclimation processes. As seen, the PSII operational quantum yield (Φ'_M_) changed in close relationship with light limitation to light saturation gradient (Fig. 5a). It remained high and stable under light limited conditions and it decreased when light intensity was above the saturation point. Interestingly, that tendency was similar for all studied species regardless of their taxonomic groups and despite different average Φ'_M_ or pigment composition. It also indicates that under light limitation, phytoplankton optimized light utilisation through high PSII quantum yield, while other biochemical or physiological factors became limiting in draining electrons under high light [9], [10]. Concomitantly to these changes, we observed an increase of NPQ and UQF_REL_ above light saturation. Non-photochemical quenching and associated processes allowed the dissipation of excess energy and alleviated the excitation pressure on PSI and PSII [54]. The unquenched fluorescence reflects the redox state of the electron transport chain [34] and the high values obtained above light saturation indicates that the PSI and/or other electron sinks were less efficient to drain electrons under high light compared to low light conditions. This lower capacity to drain electrons from PSII may be induced by PSII:PSI energy imbalance or by a lack of available reductants (NADP+ and ADP) [10], [18], [52]. Finally, the variations observed for Φ'_M_ were also reflected in the quantum requirement (QR) which remained close to the theoretical value of 8 photons per O_2_ molecule evolved with 13.4(±1.4 mol e mol O_2_^−1^) for chlorophytes, bacillariophytes and cryptophyte under light limiting conditions. However, for the cyanophytes the average QR was higher (19.4±2.0 mol e mol O_2_^−1^) indicating that this group was less efficient to convert light energy into chemical energy. Under saturating conditions, QR increased for all species to more than 30(>50 in cyanophytes) indicating a lower photosynthetic efficiency compared to low light conditions. Despite the lower conversion efficiency under light saturation, phytoplankton cells were able to maintain high growth rates indicating that adjustments to their energy dissipation processes (high NPQ and UQF_rel_ and low Φ'M) under high light conditions were not disadvantageous. These differences between phytoplankton groups clearly indicate variations in photoacclimation processes, as was also observed for pigments and optical properties.

### Primary production and growth uncoupling

The oxygen production estimates calculated from a combination of chlorophyll fluorescence and biooptical method [42] and normalized to Chl *a* (P_O2_^Chl^) varied between 0 and 2.2 mmol O_2_ mg Chl *a* ^−1^ hr^−1^ for all species (Fig. 1b). This was comparable to the range reported previously for different phytoplankton species [9], [28], [42], [44], [68]. Oxygen production was lower when normalized to biovolume (P_O2_^ µm^) and this difference can be attributed to variation in the ratio of Chl *a* per biovolume specific to individual species following photoacclimation. When normalized to Chl *a*, oxygen production was informative of the photosynthetic apparatus efficiency where high values correspond to high photosynthetic efficiency. Oxygen production normalized to biovolume allows to relate photosynthetic efficiency to biomass, and therefore to the achieved growth rate. In our study, PE curves were reconstructed similarly to growth versus light curves since they were based on photosynthetic activity obtained at different growth light intensities. This method is slightly different to PE curves obtained by short term exposure to different light intensities of pre-acclimated phytoplankton [9]. Thus, our approach permits to directly estimate if there is a relationship between growth and photosynthesis when phytoplankton is acclimated to specific light conditions. Interestingly, when comparing the light intensity required to reach maximal photosynthesis for both variables (P_M_^Chl^ and P_M_^ µm^), we found that E_M_^ µm^ (287–979 µmol photons m^−2^ s^−1^) was always lower than E_M_^Chl^ (660–1376 µmol photons m^−2^ s^−1^) for all studied species (Table 2). This difference reflects a decoupling between photosynthesis and cellular investment in chlorophyll (see below). We may therefore advance that cellular investment in the photosynthetic components and Chl *a* relative to the other cellular constituents was sub optimal for the studied species and this suggests that phytoplankton cells, in our growth conditions, did not try to maximize their photosynthetic activity, otherwise E_M_^ µm^ should tend toward E_M_^Chl^. A good example of that phenomenon was observed for *A. granulata* since this diatom has the highest P_M_^Chl^ of all tested species (Fig. 1b) and thus high photosynthetic efficiency relative to cellular Chl *a* investment. However, this high photosynthetic efficiency was not reflected into a better growth rate. In fact, we found very low oxygen production on a biomass basis (P_M_^ µm^) for this species, indicating that its strategy was not to invest in photosynthetic apparatus and Chl *a* (Fig. 1b and 1c). Consequently, this species presented one of the lowest µ_d_ and µ_MAX_ values of this study despite a potential of high photosynthetic efficiency. Our findings that growth rate approached its maximal value when oxygen production per biovolume reached saturation and the absence of change of µ_d_ above photosynthesic saturation and up to maximal photosynthesis was another indication of the decoupling between cell division and photosynthesis. This can be attributed to lower Chl *a* content in high light acclimated cells and can also be caused by an increase in the respiration processes relative to photosynthesis or by accumulation of compound that were not included in our growth rate estimates such as lipids.

### Conclusions

The general response of phytoplankton to increased light intensity worked toward reducing the excitation pressure on the photosynthetic apparatus and also toward reducing their efficiency to utilize the absorbed energy. According to our results, these mechanisms induced a decoupling between photosynthesis and growth rate when light intensity was above photosynthetic saturation level indicating that photoacclimation processes do not necessarily optimize photosynthesis to maximize growth. Interestingly, all species of our study followed that tendency despite being of different functional groups (colonial, flagellated, different size) and of different phylogeny. Even if some species did reach higher growth rates in our conditions and thus, should dominate in natural environment with respect to light intensity, we cannot exclude that other environmental factors also influence the population dynamic making the outcome difficult to predict. Finally, the fact that morphologically distinct species isolated from the same community, but belonging to different phylogenic groups, were able to adjust to a wide range of light intensities (from 14 to 1079 µmol photons m^−2^ s^−1^) demonstrates the great plasticity and adaptation ability of freshwater phytoplankton to their light environment and help to understand their ubiquity in natural environment.

## References

[1] Litchman E (2003) Competition and coexistence of phytoplankton under fluctuating light: experiments with two cyanobacteria. Aquat Microb Ecol 31: 241–48.

[2] Dubinsky Z, Stambler N (2009) Photoacclimation processes in phytoplankton: mechanisms, consequences, and applications. Aquat Microb Ecol 56: 163–176.

[3] Abeliovich A, Shilo M (1972) Photooxidative death in blue-green algae. J Bacteriol 111: 682–89. 4626540 10.1128/jb.111.3.682-689.1972 PMC251340

[4] Eloff JN, Steinit Y, Shilo M (1976) Photooxidation of cyanobacteria in natural conditions. Appl Environ Microbiol 31 (1): 119–26. 10.1128/aem.31.1.119-126.1976 PMC169727 821394

[5] Gerber S, Häder DP (1995) Effects of enhanced solar irradiation on chlorophyll fluorescence and photosynthetic oxygen production of five species of phytoplankton. FEMS Microbiol Ecol 16: 33–42.

[6] Schanz F, Senn P, Dubinsky Z (1997) Light absorption by phytoplankton and the vertical light attenuation: ecological and physiological significance. Oceanogr Mar Biol Annu Rev 35: 71–95.

[7] Richardson K, Beardall J, Raven JA (1983) Adaptation of unicellular algae to irradiance: an analyses of strategies. New Phytol 93: 157–191.

[8] Falkowski PG, La Roche J (1991) Acclimation to spectral irradiance in algae. J Phycol 27: 8–14.

[9] MacIntyre HL, Kana TM, Anning T, Geider RJ (2002) Review: Photoacclimation of photosynthesis irradiance response curves and photosynthetic pigments in microalgae and cyanobacteria. J Phycol 38: 17–38.

[10] Sukenik A, Bennett J, Falkowski PG (1987) Light saturated photosynthesis limitation by electron transport or carbon fixation? Biochim Biophys Acta 891: 205–215.

[11] Fisher T, Schurtz-Swirski R, Gepstein S, Dubinsky Z (1989) Changes in the levels of ribulose-1,5-bisphosphate carboxylase/oxygenase (RUBISCO) in *Tetraedron minimum* (Chlorophyta) during light and shade adaptation. Plant Cell Physiol 30: 221–228.

[12] Herzig R, Dubinsky Z (1992) Photoacclimation, photosynthesis, and growth in phytoplankton. Isr J Bot 41: 199–212.

[13] Steiger S, Schaëfer L, Sandmann G (1999) High-light-dependent upregulation of carotenoids and their antioxidative properties in the cyanobacterium *Synechocystis* PCC6803. J Photochem Photobiol B, Biol 52: 14–18.

[14] Grossman AR, Schaefer MR, Chiang GG, Collier JL (1993) The phycobilisome, a light harvesting complex responsive to environmental conditions. Microbiol Rev 57: 725–749. 8246846 10.1128/mr.57.3.725-749.1993 PMC372933

[15] Demmig-Adams B, Adams WW (1996) The role of xanthophyll cycle carotenoids in the protection of photosynthesis. Trends Plant Sci 1: 21–26.

[16] Kana TM, Geider RJ, Critchley C (1997) Regulation of photosynthetic pigments in micro-algae by multiple environmental factors: A dynamic balance hypothesis. New Phytol 137 (4): 629–638.

[17] Barber J, Anderson B (1992) Too much of good thing: light can be bad for photosynthesis. Trends Biochem Sci 17: 61–66. 1566330 10.1016/0968-0004(92)90503-2

[18] Huner NPA, Öquist G, Sarhan F (1998) Energy balance and acclimation to light and cold. Trends Plant Sci 3 (6): 224–230.

[19] Choudhury NK, Behera RK (2001) Photoinhibition of photosynthesis: role of carotenoids in photoprotection of chloroplast constituents. Photosynthetica 39: 481–488.

[20] Johnsen G, Sakshaug E (2007) Bio-optical characteristics of PSII and PSI in 33 species (13 pigment groups) of marine phytoplankton, and the relevance for pulse-amplitude-modulated and fast-repetition-rate fluorometry. J Phycol 43: 1236–1251.

[21] Raven JA (1998) The Twelfth Tansley Lecture, Small is Beautiful: The Picophytoplankton. Funct Ecol 12 (4): 503–513.

[22] Beardall J, Allen D, Bragg J, Finkel ZV, Flynn KJ, et al (2009) *Tansley review*: Allometry and stoichiometry of unicellular, colonial and multicellular phytoplankton. New Phytol 181: 295–309. 19121029 10.1111/j.1469-8137.2008.02660.x

[23] Reynolds CS (1998) What factors influence the species composition of phytoplankton in lakes of different trophic status? Hydrobiologia 369/370: 11–26.

[24] Reynolds CS, Huszar V, Kruk C, Naselli-Flores L, Melos S (2002) Review: Towards a functional classification of the freshwater phytoplankton. J Plankton Res 24 (5): 417–428.

[25] Agusti S, Phlips EJ (1992) Light absorption by cyanobacteria: Implications of colonial growth form. Limnol Oceanogr 32: 434–441.

[26] Wilson AE, Kaul RB, Sarnelle O (2010) Growth rate consequences of coloniality in a harmful phytoplankter. PLoS One 5 (1): e8679. 20084114 10.1371/journal.pone.0008679 PMC2799676

[27] Cullen JJ, MacIntyre JG (1998) Behavior, physiology and the niche of depth-regulating phytoplankton. In: Anderson DM, Cemballa AD, Hallegraeff GM, editors. Physiological ecology of harmful algal blooms: Springer-Verlag Heidelburg. pp. 559–580.

[28] Dubinsky Z, Falkowski PG, Wyman K (1986) Light harvesting and utilization in phytoplankton. Plant Cell Physiol 27: 1335–1350.

[29] Rolland A, Bird DF, Giani A (2005) Seasonal changes in composition of the cyanobacterial community and occurrence of hepatotoxic blooms in the eastern townships, Québec, Canada. J. Plankton Res 27 (7): 683–694.

[30] Mur LR, Schreurs H (1995) Light as a selective factor in the distribution of phytoplankton species. Water Sci Technol 32 (4): 25–34.

[31] Havens KE, Phlips EJ, Cichra MF, Li B-L (1998) Light availability as a possible regulator of cyanobacteria species composition in a shallow subtropical lake. Freshw Biol 39 (3): 547–556.

[32] Liu S, Juneau P, Qiu B-S (2012) Effects of iron on the growth and minimal fluorescence yield of three marine *Synechococcus* strains (Cyanophyceae). Phycol Res 60 (1): 61–69.

[33] Schreiber U, Schliwa U, Bilger W (1986) Continuous recording of photochemical and non-photochemical chlorophyll fluorescence quenching with a new type of modulation fluorometer. Photosynth Res 10: 51–62. 24435276 10.1007/BF00024185

[34] Juneau P, Green BR, Harrison PJ (2005) Simulated of Pulse-Amplitude-Modulated (PAM) fluorescence: limitations of some PAM-parameters in studying environmental stress effects. Photosynthetica 43 (1): 75–83.

[35] Campbell D, Hurry V, Clarke AK, Gustafsson P, Öquist G (1998) Chlorophyll fluorescence analysis of cyanobacterial photosynthesis and acclimation. Microbiol Mol Biol Rev 62 (3): 667–83. 9729605 10.1128/mmbr.62.3.667-683.1998 PMC98930

[36] Ritchie RJ (2008) Universal chlorophyll equations for estimating chlorophylls a, b, c, and d and total chlorophylls in natural assemblages of photosynthetic organisms using acetone, methanol, or ethanol solvents. Photosynthetica 46 (1): 115–26.

[37] Lichtenthaler HK, Wellburn AR (1985) Determination of total carotenoids and chlorophylls a and b of leaf in different solvents. Biol Soc Trans 11: 591–592.

[38] Bennett A, Bogorad L (1973) Complementary chromatic adaptation in a filamentous blue-green alga. J Cell Biol 58: 419–35. 4199659 10.1083/jcb.58.2.419 PMC2109051

[39] Jassby AD, Platt T (1976) Mathematical formulation of the relationship between photosynthesis and light for phytoplankton. Limnol Oceanogr 21: 540–547.

[40] Zimmerman RC, Beeler SooHoo J, Kremer JN, D'Argenio DZ (1987) Evaluation of variance approximation techniques for non-linear photosynthesis-irradiance models. Marine Biol. 95: 209–215.

[41] Kopf U, Heinze J (1984) 2,7-Bis(diethylamino)phenazoxonium chloride as a quantum counter for emission measurements between 240 and 700 nM. Anal Chem 56: 1931–1935.

[42] Hancke TB, Hancke K, Johnsen G, Sakshaug E (2008) Rate of O_2_ production devrived from pulse-amplitude-modulated fluorescence: testing three biooptical approaches against measured O_2_-production rate. J Phycol 44: 803–813. 27041438 10.1111/j.1529-8817.2008.00509.x

[43] Gilbert M, Domin A, Becker A, Wilhelm C (2000) Estimation of primary productivity by chlorophyll a in vivo fluorescence in freshwater phytoplankton. Photosynthetica 38: 111–26.

[44] Ritchie RJ (2008) Fitting light saturation curves measured using modulated fluorometry. Photosynth Res 96: 201–215. 18415696 10.1007/s11120-008-9300-7

[45] Quinn P, Keough MJ (2003) Experimental design and data analysis for biologists. Cambridge press. 537 p. ISBN 0 521 00976 6.

[46] Dunnett CW (1955) A multiple comparison procedure for comparing several treatments with a control. J Am Stat Assoc 50: 1096–1121.

[47] Wetzel RG (2001) Limnology: Lake and River Ecosystems, 3rd ed.Springer-Verlag, New York, 1006 p.

[48] Falkowski PG, Raven JA (2007) Aquatic photosynthesis. 2^nd^ ed. Princeton University Press, Princeton, NJ. 484 p.

[49] Raven JA, Kübler JE (2002) New light on the scaling of metabolic rate with the size of algae (Short survey). J Phycol 38: 11–16.

[50] Fee EJ (1976) The vertical and seasonal distribution of chlorophyll in lakes of the experimental lakes areas, northwestern Ontario: implications for primary production estimates. Limnol Oceanogr 21: 767–783.

[51] Behrenfeld MJ, Prasil O, Babin M, Bruyant F (2004) In search of a physiological basis for covariations in light-limited and light saturated photosynthesis. J Phycol 40 (1): 4–25.

[52] Sonoike K, Hihara Y, Ikeuchi M (2001) Physiological significance of the regulation of photosystem stoichiometry upon high light acclimation of *Synechocystis* sp. PCC6803. Plant Cell Physiol 42 (4): 379–384. 11333308 10.1093/pcp/pce046

[53] Huner NPA, G Öquist, Melis A (2003) Photostasis in plants, green algae and cyanobacteria: The role of light harvesting antenna complexes. In: Green BR, Parson WW editors. Light-harvesting antennas in photosynthesis. Dordrecht, Kluwer Academic Publishers. pp. 402–421.

[54] Müller P, Xiao-Ping L, Niyogi KK (2001) Non-photochemical quenching. A response to excess light energy. Plant Physiol 125: 1558–566. 11299337 10.1104/pp.125.4.1558 PMC1539381

[55] Lavaud J, Rousseau B, Etienne AL (2004) General features of photoprotection by energy dissipation in planktonic diatoms (Bacillariophyceae). J Phycol 40: 130–137.

[56] Gantt E, Conti SF (1966) Granules associated with the chloroplast lamellae of *Porphyridium cruentum* . J Cell Biol 29: 423–434. 5962937 10.1083/jcb.29.3.423 PMC2106974

[57] Raps S, Wyman K, Siegelman HW, Falkowski PG (1983) Adaptation of the cyanobacterium *Microcystis aeruginosa* to light intensity. Plant Physiol 72: 829–832. 16663094 10.1104/pp.72.3.829 PMC1066329

[58] Kana TM, Glibert PM (1987) Effect of irradiances up to 2000 µE m-2 s-1 on marine *Synechococcus* WH7803. I. Growth, pigmentation, and cell composition. Deep-Sea Res 34: 479–495.

[59] Subramaniam A, Carpenter EJ, Karentz D, Falkowski PG (1999) Bio-optical properties of the marine diazothrophic cyanobacteria *Trichodesmuim* spp. I. Absorption and photosynthetic action spectra. Limnol Oceanogr 44: 608–617.

[60] Tamary E, Kiss V, Nevo R, Adam Z, Bernát G, et al (2012) Structural and functional alterations of cyanobacterial phycobilisomes induced by high-light stress. Biochim Biophys Acta 1817: 319–327. 22138629 10.1016/j.bbabio.2011.11.008

[61] Wilson A, Ajlani G, Verbavatz JM, Vass I, Kerfeld CA, et al (2006) A soluble carotenoid protein involved in phycobilisome-related energy dissipation in cyanobacteria. Plant Cell 18: 992–1007. 16531492 10.1105/tpc.105.040121 PMC1425857

[62] Karapetyan NV (2007) Non-photochemical quenching in cyanobacteria. Biochemistry (Mosc) 72 (10): 1127–1135. 18021070 10.1134/s0006297907100100

[63] Kirilovsky D, Kerfeld CA (2012) The orange carotenoid protein in photoprotection of photosystem II in cyanobacteria. Biochim Biophys Acta 1817 (1): 158–166. 21565162 10.1016/j.bbabio.2011.04.013

[64] Johnsen G, Prezelin BB, Jovine RVM (1997) Fluorescence excitation spectra and light utilization in two red tide dinoflagellates. Limnol Oceangr 42 (S. part 2) 166–177.

[65] Geider RJ, Platt T, Raven JA (1986) Size dependence of growth and photosynthesis in diatoms: a synthesis. Mar Ecol Prog Ser 30: 93–104.

[66] Kirk JTO (1986) Optical properties of picoplankton suspensions. Can Bull Fish Aquat Sci 214: 501–520.

[67] Suggett DJ, Le Floc'H E, Harris GN, Leonardos N, Geider RJ (2007) Different strategies of photoacclimation by 2 strains of *Emiliania huxleyi* (Haptophyta). J Phycol 43: 1209–1222.

[68] Falkowski PG, Dubinsky Z, Wyman K (1985) Growth-irradiance relationships in phytoplankton. Limnol Oceanogr 30: 311–321.

